# Review of Cadmium Bioaccumulation in Fish Exposed to Cadmium

**DOI:** 10.3390/toxics13010007

**Published:** 2024-12-25

**Authors:** Ju-Wook Lee, A-Hyun Jo, Yue-Jai Kang, Dain Lee, Cheol-Young Choi, Ju-Chan Kang, Jun-Hwan Kim

**Affiliations:** 1 Incheon Regional Office of National Fishery Products Quality Management Service, Incheon 22346, Republic of Korea; leejuwook84@gmail.com; 2 Department of Marine Life Science, Jeju National University, Jeju 63243, Republic of Korea; joahyun0902@naver.com; 3 Department of Aquatic Life Medicine, Kunsan National University, Gunsan 54150, Republic of Korea; kyj5088@hanmail.net; 4 Fish Genetics and Breeding Research Center, National Institute of Fisheries Science, Geoje 53334, Republic of Korea; gene419@korea.kr; 5 Division of Marine BioScience, National Korea Maritime and Ocean University, Busan 49112, Republic of Korea; 6 Department of Aquatic Life Medicine, Pukyong National University, Busan 48513, Republic of Korea; jckang@pknu.ac.kr; 7 Department of Aquatic Life Medicine, Jeju National University, Jeju 63243, Republic of Korea

**Keywords:** cadmium, circulatory toxicity, bioaccumulation, target organs

## Abstract

Cadmium (Cd) is a highly toxic substance in the aquatic ecosystem, which can represent a high risk to fish. Fish are exposed to heavy metals through waterborne and dietary pathways, some of which are absorbed by the body and can accumulate in specific tissues without being eliminated. The accumulation varies depending on several factors such as dose, exposure route, exposure time, metal types, and biological status of the fish, and environmental parameters such as DO, salinity, pH, and metal speciation. As Cd speciation occurs in the water, the amount accumulated in the fish can vary, and consuming Cd-accumulated fish can pose a risk to human health. Cd introduced into the body of fish can directly affect blood properties through the circulatory system. Cd introduced into the circulatory system of fish can reach all tissues through the blood flow, and the accumulation of specific tissues is different depending on the blood flow by the energy and oxygen demand of each tissue. Therefore, this review aimed to determine the toxic effects of Cd exposure in fish and identify indicators to assess the extent of Cd bioaccumulation toxicity in fish induced by Cd exposure.

## 1. Introduction

Cadmium (Cd) is a biotoxic element considered a priority contaminant due to its non-biodegradable/persistent characteristics, and it is a biologically non-essential metal that is very important from the ecotoxicological and toxicological points of view [1,2]. Cd exists naturally in trace amounts in the aquatic environment (0.002–0.015 μg/L in clean rivers, increasing up to 2–3 μg/L or greater in surface waters of the impacted environments), but it is particularly prevalent in the production and processing of products during industrialization, as well as in agricultural, mining, and chemical processes, where it is released or mobilized into the aquatic environment at high levels, threatening aquatic ecosystems [3,4]. Metals are classified into essential and non-essential trace elements based on their role in fish metabolism, and Cd, a non-essential metal, is highly toxic even at low concentrations in aquatic ecosystems, which is why it is classified as a hazardous substance under the Water Framework Directive (2000/60/EC) [4]. Cd is found in low levels as suspended particulate matter in water, but it can be present in high concentrations in sediments; when seawater and freshwater are warm or agitation occurs, sediments contaminated by Cd can rise into the water, which can affect aquatic organisms [5].

Cd may cause disturbances in the endocrine mechanism of fish, thereby damaging the reproductive activity and development of fish; according to several reports, Cd is known to have toxic effects on sperm cell function, steroid levels in females, vitellogenesis, estrogen receptor expression, and male/female gametogenesis in fish; it poses a health risk to humans who consume fish contaminated with Cd [6]. Low-level Cd exposure has been reported to impair biochemical and physiological functions, along with disorders in osmotic and ionic regulation, cell damage, and several enzymatic activity changes in fish, and chronic exposure to Cd in fish can cause dysfunction of tissues such as kidney and liver, which can lead to growth inhibition, oxidative stress, immune damage, and even death [7]. Cd exposure affects hypothalamic–pituitary–interrenal axis signals in fish, leading to endocrine disruption. Seawater fish continuously consume seawater to maintain homeostasis at high osmolality in the environment, so exposure to Cd may increase, causing osmotic dysregulation. On the other hand, in freshwater fish, exposure to Cd induces toxicity through inhibition of gill calcium absorption [8]. Toxic effects of Cd exposure in fish can appear in various ways, and the toxic physiology in fish caused by Cd exposure is considered through a multifaceted approach in this review, thereby presenting the criteria for establishing Cd toxicity indicators in the future.

## 2. Bioaccumulation

### 2.1. Bioaccumulation Patterns

Metals can potentially reach toxic concentration levels in aquatic environments, and when metals entering the fish body exceed excretion levels, they can cause accumulation in certain organs [9]. Determination of metals in fish following metal exposure is very important because metals in water can accumulate in the tissues of aquatic organisms and act as a hazard to human health through the food chain [10]. When Cd-contaminated fish are ingested and digested/absorbed by humans, some heavy metals are excreted, but some also accumulate in many human tissues, leading to toxicity/poisoning symptoms. When the concentration of metals introduced into the fish body exceeds the limiting load, it can cause homeostasis disturbance and disruption, which can have acute or chronic toxic effects on fish. Metal accumulation in fish, according to metal exposure, can appear differently for each tissue, and the prominent accumulation pattern in a specific tissue is a major target organ where toxicity is expressed and can be used for toxicity analysis [11]. Among metals, Cd is one of the most threatening heavy metals to the human body (Pb > Cd > Ni > Zn > Cu > Mn, Agency for Toxic Substances and Disease Registry), and it is a hazardous element that has toxic effects including teratogenesis, carcinogenesis, deformation, mutagenesis, and breakdowns of organs on humans [12].

The accumulation of these heavy metals varies depending on several factors, such as concentrations in the environment, exposure route, exposure time, biological status of the fish (sex, stage of development), and environmental parameters (water temperature, salinity, pH, dissolved oxygen, metal speciation, etc.) [13]. For example, exposure of Cd to fish has a stronger effect on freshwater fish compared to marine species, because it is inversely proportional to sat concentration [14]. Cd can accumulate in fish via direct absorption or biomagnification, and depending on the organism, the half-life of Cd can reach 30 to 70 years, which can cause significant tissue and organ damage; biomagnification refers to an increase in the concentration of toxic substances in the tissues of an organism through the food chain [2]. Metals present in water may be introduced to fish through metal ions dissolved in water or food with metal accumulation, and the accumulation pattern may appear differently depending on the inflow route [15]. In addition, Cd bioaccumulation in fish tissues is influenced by the speciation of Cd compounds in water, which varies depending on the organic carbon content and pH in the environment; Cd speciation in water involves the formation of complexes or different ions from water-soluble inorganic compounds such as Cd halides and Cd sulfate [16]. Cyanobacterial cells or debris with a high metal concentration can be ingested by small fish through food, and it may contribute to an increase in metal concentration in small fish [17]. When Cd enters the fish body, it is transferred to the liver or skeleton through the bloodstream; Cd deposited in the fish body affects reproduction, inducing continuous toxicity [18]. High concentrations of Cd accumulation in fish tissues induce various physiological changes such as enzyme activity, ion regulation, tissue morphology, and skeletal malformations [19].

**Figure 1:**
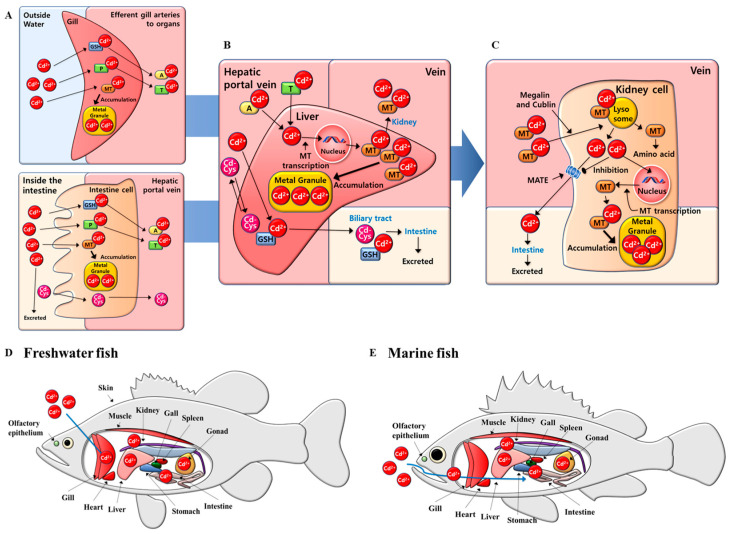
Schematic diagram of the mechanisms involved in cadmium uptake in major target organs in fish (GSH: Glutathione, MT: Metallothionein, P: Ca^2+^-binding protein, A: Albumin, T: Transferrin, Cd–Cys: Cd–Cys/Peptides, MATE: Multidrug and toxin extrusion protein). (**A**) absorption mechanism in fish body of cadmium through gills and intestines and intracellular accumulation mechanism by metallothionein. (**B**) inducing Cd-MT formation and Cd accumulation in cytoplasm through promotion of MT synthesis of Cd and Cd-albumin. (**C**) reabsorption Mechanism of Cd through proximal tubules in the kidney. (**D**) mechanism of Cd accumulation in freshwater fish. (**E**) mechanism of Cd accumulation in marine fish.2.2. Bioaccumulation Mechanisms.

A schematic representation of the mechanisms involved in Cd uptake in major target organs in fish is demonstrated in Figure 1. Accumulation patterns following Cd exposure may differ between freshwater and marine fish. Toxicity following acute Cd exposure in freshwater fish includes inhibition of calcium absorption by gill tissues; freshwater fish actively absorb Na^+^ and Cl^−^ from their gills to save ions, whereas in marine fish, Na^+^ and Cl^−^ are actively excreted to control osmotic pressure and maintain homeostasis [20]. Intestinal tissue accumulation due to Cd exposure may be more pronounced in environments with high salinity, such as brackish and seawater conditions [21]. Although fish tend to accumulate metals in specific tissues through water and food, they tend to accumulate in major metabolically active tissues such as the liver, kidney, spleen, and gills regardless of intake routes [22].

Cd in the water (divalent cation) is absorbed into the fish body through the gills and intestines, and it is accumulated in the cells by metallothionein (MT) (Figure 1A); (i) Cd introduced through the gills enters the cell through divalent metal transporter-1 (DMT1) and epithelial Ca^2+^ channel (ECaC) of gill epithelium and acts as a Ca^2+^ analog to antagonize Ca^2+^ [23,24]. Cd introduced into the body is released from the gill cell through the high-affinity Ca^2+^-ATPase and Na^+^/Ca^2+^ exchanger channels, and it is bound to binding proteins such as albumin and MT, thereby moving to the blood [25]. (ii) Cd is taken up into the intestine by metal transporters such as the ferrous iron transporter (DMT1), Zn/Fe-regulated transporter (ZRT/IRT)-related protein 14 (ZIP14), and transient receptor potential receptor (TRPV6) [26,27]. Additionally, Cd (Cd–Cys) conjugated to cysteine or cysteine-containing oligopeptides can be absorbed because the intestinal epithelium is very rich in amino acids and small peptide transporters [28]. Cd is released from the intestinal cell through a Na^+^/Ca^2+^ exchange mechanism, and a membrane transporter, such as metal transport protein-1 (MTP1), binds to albumin or other polymeric proteins, thereby transporting them to the liver through the hepatic portal system [29]. Cd in the intestine is partially excreted in the feces, which is also transported directly from the intestinal mucosa to the kidney in the form of Cd–MT [26].

Cd and Cd–albumin introduced into hepatocytes through DMT1, ECaC, and amino acid and/or peptide transporters promote MT synthesis, thereby forming more Cd–MT in the cell cytoplasm and causing Cd accumulation (Figure 1B) [30,31]. In the liver, some Cd–MT, and Cd–Cys migrate to the bile and into the intestine through bile to be excreted from the body. The Cd–MT complex and Cd–Cys are released from the liver through membrane transporters such as MTP1 and amino acid and/or small peptide transporters, and the Cd–MT complex released from hepatocytes moves through the blood to the kidneys [24,30]. In the kidney, Cd is filtered from the glomeruli and reabsorbed in the proximal tubules (Figure 1C). Cd is absorbed through receptors such as DMT1, Zir, Irt-like protein 8 (ZIP8), Zir, Irt-like protein 10 (ZIP10), and voltage-dependent calcium channels (VDCC) and endocytosis by megalin and cublin [32,33]. The Cd–MT complex is introduced by the receptor, and endocytosis fuses with the lysosome to release Cd^2+^, and MT is degraded into amino acids [34]. Cd^2+^ released into the cell promotes MT synthesis and forms a Cd–MT complex to decrease or increase the accumulation of Cd^2+^ [35]. Kidney cells remove cationic heavy metals such as Cd through multidrug and toxin extrusion protein (MATE), but increased intracellular Cd^2+^ inhibits MATE [36]. Cd can be introduced into the fish body through the fish skin and olfactory epithelium, in addition to the gill and intestine, although Cd^2+^ entering from the skin is very small compared to the gill and intestine (Figure 1D,E). Cd^2+^ enters the olfactory epithelium through Ca^2+^ transporter channels such as ECaC, and it binds to the MT and accumulates in the olfactory bulb along the olfactory nerve [37]. However, it is difficult to accumulate in the brain or central nervous system, because Cd cannot pass through the blood–brain barrier (BBB) or synapses in the olfactory bulb [38].

### 2.2. Bioaccumulation Patterns in the Experimental Condition

Bioaccumulation patterns in the specific tissues of fish exposed to Cd under experimental conditions are demonstrated in Table 1. Ref. [39] reported that waterborne Cd exposure induced significant accumulation in liver and kidney tissues of common carp, *Cyprinus carpio*, which means that Cd absorbed from the gills or intestines can be transported through the circulatory system to tissues, causing high accumulation. Ref. [40] showed that waterborne Cd exposure induced accumulation of *C. carpio* in major organs, and the level of Cd accumulation was intestinal > kidney > liver > gills > muscle. Ref. [41] reported that Cd waterborne exposure induced accumulation in major organs of *C. auratus* (3 days: Gill > Liver > Muscle, 12 days: Liver > Gill > Muscle). Cd ions in water come into direct contact with the gills and show high accumulation by binding in a non-specific manner to mucopolysaccharides (components of mucoproteins, which are glycoproteins) present outside the gills. After absorption of Cd by gills, blood is transported to the liver or kidney tissue for storage and metal detoxification, whereas muscle tissue induces lower accumulation. Ref. [42] reported that waterborne Cd exposure induced accumulation (Kidney > Liver > Gills > Spleen > Muscle) in major organs of the Japanese eel, *Anguilla japonica*, and the results suggest that waterborne Cd passively diffuses through gill calcium channels and accumulates in major organs. Relatively low Cd accumulation in the spleen implies a high capacity for metal removal due to higher MT expression in the spleen. Low accumulation of Cd in muscle was observed, which is significant since humans primarily ingest muscle tissue (commonly referred to as “meat”) rather than organs, where Cd levels are typically higher in *A. japonica*.

Ref. [4] reported that waterborne Cd exposure induced a marked accumulation in intestinal and liver tissues of zebrafish, *Danio rerio*, and the results suggest that Cd, which enters the fish body through the intestine and gill tissues, moves to the liver for metal storage and detoxification. Ref. [43] reported that waterborne Cd exposure causes accumulation of major organs (Liver > Kidney > Gonad > Gills > Muscle) in *Oreochromis niloticus*; high accumulation was induced in tissues with high physiological activity, whereas muscle tissue showed relatively low accumulation. Ref. [44] reported that water-induced Cd exposure resulted in accumulation in the major organs (Kidney > Gill > Liver) of *Oncorhynchus mykiss*, confirming that the kidney was the main target organ with the highest accumulation of tissue. Ref. [45] reported that dietary Cd exposure induces accumulation of major organs (Gill > Liver > Kidney) in *O. mykiss*, and the highest Cd accumulation in the gills was due to specific transport proteins in the gills. Ref. [46] reported that waterborne Cd exposure resulted in a significant accumulation of *P. olivaceus* in major tissues (20 days: Gill > Intestine > Liver > Muscle; 30 days: Intestine > Gill > Liver > Muscle). Cd accumulation in the gills is due to an increase in the amount of mucus on the gill surfaces during metal exposure. Because Cd exposure in fish leads to detoxification and excretion through induction of metal-binding proteins such as MT in kidney and liver tissues, Cd accumulation in these organs is more pronounced. The high accumulation of Cd in the intestinal tissue of marine fish is due to the large amount of water entering the intestines of marine fish compared to freshwater fish. Ref. [47] reported that dietary Cd exposure resulted in the accumulation of major tissues (Liver > Intestine > Gill) in Crescent Grunter, *Terapon jarbua*. The major accumulation of hepatic tissue is due to the sustained transport of Cd from food exposure in the gastrointestinal tract through the hepatic portal vein system. Cd absorbed through fish feed accumulates in the intestinal tissues, affecting intestinal absorption and osmotic homeostasis, and thus seawater entering the fish’s body can also contribute to Cd accumulation [48].

**Table 1:** Bioaccumulation patterns of fish exposed to cadmium.

Exposure Route		Fish Species	Cd Concentration	Exposure Time	Response Concentration	Reference
Freshwater	Waterborne exposure	*Danio rerio*	10 μg/L	21 days	Intestine > Liver	[4]
		*Cyprinus carpio* L.	0.5 mg/L	2 weeks	Liver > Kidney	[39]
			3.0 ± 0.4 μg/L	1 day	Gill	[49]
				3 days		
				7 days		
			53, 433 μg/L	127 days	Kidney > Liver > Muscle	[50]
		*Carassius auratus gibelio*	1, 2 mg/L	2 weeks	Intestine > Kidney > Liver > Gill > Muscle	[40]
				4 weeks		
			100, 500 μg/L	2 weeks	Intestine	[51]
		*Carassius auratus*	0.1 mg/L	3 days	Gill > Liver > Muscle	[41]
				12 days	Liver > Gill > Muscle	
		*Anguilla japonica*	0.15, 0.30, 0.61, 1.83, 3.08, 3.67 mg/L	96 h	Kidney > Liver > Gills > Spleen > Muscle	[42]
		*Oreochromis niloticus*	0.5, 1.0, 1.5, 2.0, 2.5, 3.0 mg/L	1 week	Liver > Kidney > Gonad > Gills > Muscle	[43]
				2 weeks		
				3 weeks		
			0.4, 2.0, 4.1, 7.5 mg/L	21 days	Liver	[52]
		*Oncorhynchus mykiss*	3 μg/L	20 days	Kidney > Gill ≃ Liver	[44]
				30 days	Kidney > Gill > Liver	
			3, 10 μg/L	30 days	Gill > Liver	[53]
			2 μg/L	1 week	Gill > Kidney > Liver	[54]
		*Pelteobagrus fulvidraco*	50, 200 μg/L	8 weeks	Intestine	[55]
		*Tilapia nilotica*	0.1, 1 mg/L	10 days	Liver > Gill > Muscle	[56]
	Dietary exposure	*Oncorhynchus mykiss*	298.88 ± 19.69 μg Cd/g	15 days	Gill > Liver ≃ Kidney	[45]
				30 days	Gill > Liver > Kidney	
Seawater	Waterborne exposure	*Paralichthys olivaceus*	0, 10, 50, 100 μg/L	20 days	Gill > Intestine > Liver > Muscle	[46]
				30 days	Intestine > Gill > Liver > Muscle	
		*Terapon jarbua*	0.6 mg/L	14 days	Gill > Liver ≃ Intestine	[47]
				28 days	Liver > Intestine > Gill	
		*Sparus aurata*	1 mg/L	2 days	Liver > Muscle	[57]
				10 days		
				30 days		
	Dietary exposure	*Terapon jarbua*	27.1, 64.8 μg/g dw	14 days	Liver > Intestine > Gill	[47]
				28 days		

### 2.3. Bioaccumulation Patterns in the Field Monitoring

Bioaccumulation patterns in the specific tissues of fish exposed to Cd under field environments are demonstrated in Table 2. Ref. [58] argued that naturally occurring Cd can migrate from minerals to water bodies under the influence of acidic atmospheric precipitation and that there was an increase in Cd concentration with decreasing pH in Swedish rivers. Cd naturally present in the waters of Pechora of Russia caused significant tissue accumulation of fish species such as European whitefish, *Coregonus lavaretus,* and northern pike, *Esox lucius*, with the highest levels found in the kidney and liver. Ref. [6] argued that industrialization causes seasonal accumulation of various metals, including Cd, in Meiliang Bay and Taihu Lake, China. As a result of monitoring fish species such as *C. carpio* and Korean bullhead, *Pseudobagrus fulvidraco*, significant accumulation occurred in the kidneys and gills in *C. carpio* and in the intestine and kidney (summer) and liver and intestine (winter) in *P. fulvidraco*. In the results of the study, the tissue accumulation patterns of the two fish species were different. The affinity for metal uptake is determined by various interaction factors, such as ecological needs, feeding behavior, and sediment pollution gradients, showing a significant correlation between fish species. Ref. [59] suggests that Cd may cause high accumulation due to the large surface area of gills, which are favorable for Cd absorption and bioaccumulation, and they argued that the Cd accumulation of fish in aquatic ecosystems could be highly correlated with fish age, as accumulation in the liver is not readily excreted. Ref. [60] reported that demersal fish such as bull shark, *Carcharhinus leucas*, Blackfin barracuda, *Sphyraena qenie*, Australian Halibut, *Psettodes erumei*, largehead hairtail, *Trichiurus lepturus,* and tigertooth croaker, *Otolithes ruber* had higher metal accumulation than pelagic fish such as streaked Spanish mackerel, *Scomberomorus lineolatus* and Common Hairfin Anchovy, *Setipinna tenuifilis*, and they argued that this was due to direct contact with seafloor sediments with high levels of metals, interactions with benthic organisms, and increased uptake from benthic predators.

### 2.4. Cadmium Toxicity Reduction Strategies

Cd is a globally recognized water pollutant that poses significant threats to both aquatic ecosystems and human health. When released into aquatic environments, Cd can be absorbed by fish and subsequently bioaccumulate through the food chain, ultimately endangering human health [75]. Cd can persistently accumulate in the bodies of aquatic organisms and humans, potentially leading to greater risks over time due to the absence of metabolic substances that can break it down and the insufficient presence of chelating agents [76]. In particular, Cd induces oxidative stress in the human body and disrupts the antioxidant system, affecting the immune system, which can lead to cancer by persistent DNA mutations or damage. As a result, it is classified as a Group I carcinogen by the International Agency for Research on Cancer (IARC) [77]. Chronic Cd exposure can cause respiratory damage, chronic obstructive pulmonary disease (COPD), chronic rhinitis, and Itai-itai disease, with its bioavailability increasing with age, further heightening health risks in older individuals [78]. Therefore, Cd toxicity impacts various biological systems in both aquatic ecosystems and humans, highlighting the need for effective strategies to monitor and reduce Cd in environmental contamination.

**Figure 2:**
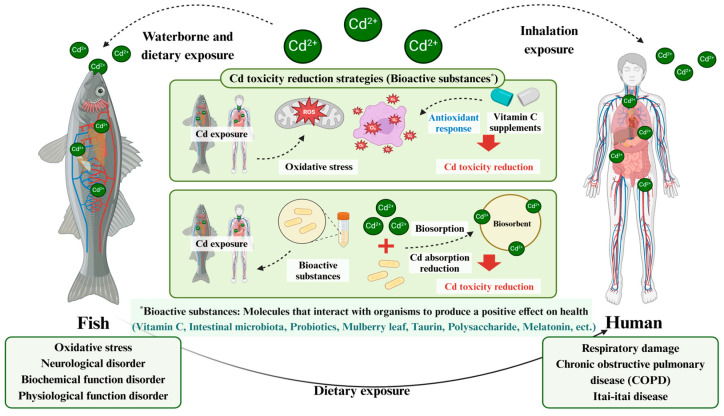
Schematic diagram of Cd toxicity reduction strategies in bioactive substances in impacts on fish and human health. Created in BioRender. Created with BioRender.com (https://BioRender.com/g81u057, accessed on 19 December 2024).

Cd toxicity reduction strategies in bioactive substances of fish exposed to Cd under experimental environments are demonstrated in Table 3 and Figure 2. Probiotics have shown significant potential in reducing Cd toxicity. Ref. [79] reported that supplementation with the probiotic *Lactobacillus plantarum* significantly reduced Cd accumulation in the spleen, kidney, gills, and muscle tissues of *O. niloticus*, which suggested that dietary supplementation with *L. plantarum* facilitates Cd with *L. plantarum* excretion in fish, enhancing its elimination from the body. Similarly, *Bacillus coagulans* demonstrated the ability to bind Cd in water, thereby reducing Cd absorption in the gills and intestines of *C. carpio* and lowering Cd concentrations in the liver and kidney [7]. Antioxidant-rich substances have also been effective in mitigating Cd accumulation. Ref. [80] reported that supplementation with the mulberry leaf significantly reduced Cd accumulation in the Liver and muscle tissues of the rare minnow, *Gobiocypris rarus,* due to the antioxidant capacity of the mulberry leaf. Ascorbic acid (vitamin C) showed chelating properties, significantly decreasing Cd accumulation in multiple tissues, including the kidney, liver, and gills of *Platichthys stellatus* and *C. carpio*. Vitamin C was further observed to compete with Cd for sulfhydryl-binding sites on metallothionein, reducing tissue retention [81,82].

Other bioactive agents, such as taurine and polysaccharides, have demonstrated tissue-specific effects. Ref. [83] reported that supplementation with taurine significantly reduced Cd accumulation in the muscle tissue of red sea bream, *Pagrus major*. Melatonin, known for its antioxidant properties, mitigated Cd toxicity by neutralizing reactive oxygen species, particularly in the muscle tissues of *C. gibelio* [84]. Ref. [85] reported that supplementation with the polysaccharide, *Ganoderma lucidum* significantly reduced Cd accumulation in the muscle, brain, and liver tissues of *C*. *carpio*, which indicated that the polysaccharide was used as a ligand for metal ions, reducing the accumulation of tissues because it could effectively adsorb or chelate Cd^2+^. Intestinal microbiota, such as *Bacillus cereus*, also contributed to Cd reduction. Ref. [40] reported that supplementation with the intestinal microbiota, *Bacillus cereus,* significantly reduced Cd accumulation in the intestine and gills of gibel carp, *C. gibelio*, which suggested that the cell wall of *B. cereus* has high peptidoglycan and teichoic acid content, which can reduce the concentration of lead accumulation in the body by adsorbing Cd from the intestines and gills of fish. Zinc-enriched *B. cereus* further enhanced Cd reduction across various tissues, including the kidney, gut, liver, muscle, and gills of mirror carp, *Cyprinus carpio nudus* [86]. These findings underscore the importance of exploring dietary supplements and bioactive agents as practical strategies for reducing Cd. In addition to these strategies, it is crucial to address the source of Cd contamination by reducing its release into the environment. Proper recycling of Cd-containing products, especially batteries, and minimizing industrial discharges are essential steps to limit Cd pollution in aquatic ecosystems. By preventing Cd exposure in the first place, the risk of bioaccumulation and its harmful effects on both aquatic organisms and humans can be significantly reduced.

**Table 3:** Cd toxicity reduction strategies in bioactive substances of fish exposed to cadmium.

Exposure Route		Fish Species	Cd Concentration	Exposure Time	Bioactive Substance	Exposure Concentration	Mitigation Concentration	Reference
Freshwater	Waterborne exposure	*Oreochromis niloticus*	1 mg/L	4 weeks	*Lactobacillus plantarum*	10^8^ CFU/g	10^8^ CFU/g	[79]
		*Gobiocypris rarus*	1, 10 μg/L	28 days	Mulberry leaf	10, 30 g/kg dry weight	10, 30 g/kg dry weight	[80]
		*Carassius auratus gibelio*	1, 2 mg/L	2 and 4 weeks	*Bacillus cereus*	10^8^ cfu/g	10^8^ cfu/g	[40]
			0.4, 4 mg/L	7 and 13 weeks	Melatonin	-	-	[84]
		*Cyprinus carpio*	0.5 mg/L	60 days	*Bacillus coagulans*	10^8^ cfu/g	10^8^ cfu/g	[7]
			1, 2 mg/L	4 and 8 weeks	Ascorbic acid	150, 300 mg/kg	150, 300 mg/kg	[82]
			0.5 mg/L	2 and 4 weeks	*Ganoderma lucidum*	2, 4 g/kg	2, 4 g/kg	[85]
		*Cyprinus carpio nudus*	1 mg/L	15 and 30 days	*Bacillus cereus*	30 mg/kg	30 mg/kg	[86]
Seawater	Waterborne exposure	*Pagrus major*	0.2 mg/L	68 days	Taurine	0.5, 5%	5%	[83]
	Dietary exposure	*Platichthys stellatus*	0, 40, 80 mg/kg	2 and 4 weeks	Ascorbic acid	500, 1000 mg/kg	500, 1000 mg/kg	[81]

## 3. Conclusions

Cd introduced into the fish circulatory system is accumulated in the major tissues, with a more pronounced tendency to accumulate in physiologically active tissues such as the liver, kidneys, and gills; the accumulation tendency was also affected by habitat (freshwater or seawater) and exposure route (waterborne exposure or dietary exposure). Cd in the water can be absorbed into the fish body, promoting MT synthesis and forming Cd–MT complexes in cells to change the accumulation of Cd. Therefore, Cd exposure causes accumulation in specific tissues, and the major tissues can be used as target organs for evaluating Cd toxicity. In conclusion, the bioaccumulation presented in this review can serve as a valuable reference for assessing the toxic effects of Cd on aquatic ecosystems. Furthermore, to mitigate the risks of Cd exposure, it is essential to address its environmental sources, including proper recycling and waste management. In the future, given the toxicity of Cd to both aquatic ecosystems and human health, further research is needed to develop and optimize effective strategies for mitigating Cd contamination.

## Figures and Tables

**Table 2:** Cadmium bioaccumulation patterns of fish in the field.

Country	Location	Fish Species	Cd Concentration in Field	Accumulation Profile	Reference
Russia	Pechora	*Coregonus lavaretus*	0.08 ± 0.07 μg/L	Kidney > Liver > Gill > Muscle	[58]
		*Esox lucius*		Kidney > Liver > Gill > Muscle	
	Severnaya Dvina	*Coregonus lavaretus*	0.07 ± 0.05 μg/L	Kidney > Gill > Liver > Muscle	
		*Abramis brama*		Liver > Kidney > Gill > Muscle	
		*Esox lucius*		Kidney > Liver > Gill ≃ Muscle	
	Volga	*Abramis brama*	0.13 ± 0.04 μg/L	Kidney > Liver > Gill > Muscle	
		*Esox lucius*		Gill > Kidney > Liver > Muscle	
		*Abramis brama*	0.12 ± 0.11 μg/L	Kidney > Liver > Gill > Muscle	
		*Perca fluviatilis*		Kidney > Liver > Gill > Muscle	
		*Abramis brama*	0.13 ± 0.07 μg/L	Kidney > Liver > Gill > Muscle	
		*Perca fluviatilis*		Liver > Kidney > Gill > Muscle	
		*Esox lucius*		Gill > Kidney > Liver > Muscle	
	Kola Mountain	*Salmo trutta*	<0.05 μg/L	Kidney > Liver > Gill > Muscle	
	Kola Tundra				
	Kola Taiga				
	Pirenga	*Coregonus lavaretus*	0.08 ± 0.05 μg/L	Kidney > Liver > Gill > Muscle	
		*Perca fluviatilis*		Liver > Kidney > Gill > Muscle	
		*Esox lucius*		Kidney > Gill > Muscle > Liver	
	Arhangelsk Taiga	*Perca fluviatilis*	0.06 ± 0.05 μg/L	Kidney > Liver > Gill > Muscle	
		*Esox lucius*		Liver > Gill > Muscle	
	Karelia Taiga	*Perca fluviatilis*	0.10 ± 0.01 μg/L	Liver > Kidney > Gill > Muscle	
		*Esox lucius*		Kidney > Gill > Liver > Muscle	
India	Ganga River	*Cirrhinus mrigala*	Highest: 0.85 mg/L, Lowest: 0.54 mg/L	Liver > Gill > Muscle	[57]
		*Cirrhinus reba*		Liver > Gill > Muscle	
		*Catla catla*		Liver > Gill > Muscle	
		*Labeo rohita*		Gill > Liver > Muscle	
		*Crossocheilus latius*		Liver > Gill > Muscle	
		*Clupisoma garua*		Liver > Gill > Muscle	
		*Mystus tengara*		Liver > Gill > Muscle	
	Ramganga river	*Channa punctatus*	0.101 ± 0.05 μg/L	Liver > Kidney	[61]
	Kollidam River	*Mystus vittatus*	-	Muscle > Liver > Kidney > Intestine > Gill	[62]
		*Tilapia mossambica*		Liver > Gill > Muscle > Intestine > Kidney	
		*Ctenopharyngodon idella*		Gill > Liver > Kidney > Muscle > Intestine	
		*Saurida undosquamis*		Kidney = Muscle > Liver > Gill > Intestine	
		*Heteropneustus fossilis*		Intestine > Liver > Kidney > Gill > Muscle	
China	Taihu Lake	*Cyprinus carpio*	-	Summer: Kidney > Gill > Muscle > Intestine > Liver	[6]
				Winter: Kidney > Gill > Intestine > Muscle > Liver	
		*Pseudobagrus fulvidraco*	-	Summer: Intestine > Kidney > Muscle > Liver > Gill	
				Winter: Liver > Intestine > Muscle > Kidney > Gill	
	Chengdu polycultureponds	*Carassius carassius*	-	Liver > Muscle	[63]
		*Cyprinus carpio*			
		*Ctenopharyngodon idella*			
Nigeria	Geriyo Lake	*Clarias anguillaris*	-	Dry: Flesh > Gill > Liver	[59]
				Wet: Flesh > Liver > Gill	
		*Heterotis niloticus*	-	Dry: Gill > Flesh > Liver	
				Wet: Liver > Gill > Flesh	
		*Tilapia zilli*	-	Dry: Gill > Flesh > Liver	
				Wet: Gill > Liver > Flesh	
	Cika Koshi reservoir	*Oreochromis niloticus*	0.64 ± 0.10 mg/L	Liver > Flesh > Gill	[64]
		*Clarias gariepinus*		Gill > Flesh > Liver	
		*Bagrus bayad*		Liver > Flesh > Gill	
	Aiba Reservoir	*Marcusenius senegalensis*	-	Liver > Kidney > Gill > Muscle > Intestine	[65]
		*Labeo senegalensis*		Liver > Kidney > Gill ≈ Muscle	
		*Hepsetus odoe*		Liver > Kidney > Gill ≈ Muscle > Intestine	
		*Chrysichthys auratus*		Kidney > Liver > Gill > Muscle > Intestine	
		*Chrysichthys nigrodigitatus*		Kidney > Gill > Liver > Muscle	
		*Clarias ebriensis*		Kidney > Liver > Gill > Intestine > Muscle	
		*Clarias macromystax*		Gill > Intestine > Muscle	
		*Channa obscura*		Kidney > Gill > Muscle	
		*Tilapia zillii*		Kidney > Liver > Muscle ≈ Gill	
		*Sarotherodon galilaeus*		Kidney > Liver > Gill > Muscle	
		*Oreochromis niloticus*		Kidney > Liver > Gill ≈ Muscle	
	Nun River	*Synodontis clarias*	0.001 ± 0.000 mg/L	Bone > Muscle	[66]
		*Cithrinus citharus*			
		*Synodontis clarias*			
		*Cithrinus citharus*			
		*Synodontis clarias*			
		*Cithrinus citharus*			
		*Synodontis clarias*			
		*Cithrinus citharus*			
		*Synodontis clarias*			
		*Cithrinus citharus*			
West Africa	Bandama River	*Chrysichthys nigrodigitatus*	-	Muscle > Liver > Kidney	[67]
		*Sarotherodon melanotheron*		Liver > Kidney > Muscle	
	Comoé River	*Chrysichthys nigrodigitatus*	-	Liver > Kidney > Muscle	
		*Sarotherodon melanotheron*		Liver > Kidney > Muscle	
	Bia River	*Chrysichthys nigrodigitatus*	-	Kidney > Liver > Muscle	
		*Sarotherodon melanotheron*		Liver > Kidney > Muscle	
Malaysia	Miri Coast	*Carcharhinus leucas*	-	Gonad > Muscle ≈ Gill > Liver	[60]
		*Scomberomorus lineolatus*		Gill > Gonad > Muscle > Liver	
		*Sphyraena qenie*		Gill > Gonad > Muscle > Liver	
		*Setipinna tenuifilis*		Gill > Gonad > Muscle	
		*Psettodes erumei*		Liver > Gill > Muscle > Gonad	
		*Trichiurus lepturus*		Muscle > Gill	
		*Otolithes ruber*		Gonad > Muscle	
	Pulau Ketam, Port Klang coast	*Lates calcarifer*	0.25 ± 0.01 mg/L	Liver > Muscle	[68]
		*Lutjanus campechanus*			
		*Lutjanus griseus*			
Brazil	Aquidauana River	*Hypostomus regani*	-	Liver > Muscle	[69]
		*Prochilodus lineatus*			
		*Brycon hilarii*			
		*Mylossoma duriventre*			
Pakistan	Shah Alam River	*Mastacembelus armatus*	0.05 ± 0.02 mg/L	Kidney > Liver > Gill > Muscle ≈ Skin	[70]
		*Clupisoma naziri*		Muscle > Liver > Kidney > Skin > Gill	
Tunisia	Gulf of Gabes	*Salaria basilisca*	0.61 ± 0.02 μg/L	Liver > Gill	[71]
		*Zosterisessor ophiocephalus*		Liver > Gill	
		*Solea vulgaris*		Gill > Liver	
		*Salaria basilisca*	0.26 ± 0.03 μg/L	Liver > Gill	
		*Zosterisessor ophiocephalus*		Liver > Gill	
		*Solea vulgaris*		Gill > Liver	
		*Salaria basilisca*	0.03 ± 0.01 μg/L	Liver > Gill	
		*Zosterisessor ophiocephalus*		Liver ≈ Gill	
		*Solea vulgaris*		Gill > Liver	
Italy	Faro lake	*Mugil cephalus*	0.4 ± 0.02 μg/L	Liver > Gill	[71]
Iran	Caspian Sea	*Chelon auratus*	0.3 ± 0.01 μg/L	Liver > Gill > Muscle	[72]
		*Platycephalus indicus*	-	Small size: Liver > Kidney > Gill > Muscle	[73]
				Big size: Liver > Kidney > Gill > Muscle	
		*Pampus argenteus*		Small size: Liver > Kidney > Gill > Muscle	
				Big size: Liver > Kidney > Gill > Muscle	
Bangladesh	Kawran Bazar fish market	*Labeo rohita*	-	Gill > Liver > Muscle > Kidney	[74]
		*Gibelion catla*		Gonad > Muscle > Kidney > Gill > Liver	
		*Pangasius hypophthalmus*		Liver > Gill > Muscle > Kidney	

## Data Availability

Not applicable.
